# Healthy Vision Month — May 2014

**Published:** 2014-05-09

May is Healthy Vision Month, a national observance to promote prevention and early detection of eye diseases to reduce avoidable vision impairment, defined as the best-corrected visual acuity less than 20/40 in the better-seeing eye (1). CDC’s Vision Health Initiative partners with the National Eye Institute’s National Eye Health Education Program in encouraging everyone to make vision and eye health a priority.

Early detection, timely treatment, and use of protective eyewear are the best ways to keep eyes healthy and to prevent or delay vision impairment. In 2010, approximately 4.2 million persons in the United States aged ≥40 years had vision impairment (2). Vision impairment is the third most common chronic condition among those aged ≤17 years, the ninth most common for those aged 50–64 years, and the seventh most common for persons aged ≥65 years (3,4). Vision impairment is associated with an increased risk for falls, fall-related injuries, depression, and reduced overall health and quality of life (5–7).

Many common eye diseases have no early signs; therefore, regular, comprehensive dilated eye examinations to detect and treat vision problems and eye diseases early are recommended for all persons aged ≥65 years and for younger persons with diabetes or risk factors for glaucoma (8). Additional information about activities to promote prevention, early detection, and treatment of eye diseases and vision impairment is available at http://www.cdc.gov/visionhealth and http://www.nei.nih.gov/healthyeyes.
